# Quantitative iron–neuromelanin MRI associates with motor severity in Parkinson's disease and matches radiological disease classification

**DOI:** 10.3389/fnagi.2023.1287917

**Published:** 2023-11-27

**Authors:** Septian Hartono, Robert Chun Chen, Thomas Welton, An Sen Tan, Weiling Lee, Peik Yen Teh, Celeste Chen, Wenlu Hou, Wei Ping Tham, Ee Wei Lim, Kumar M. Prakash, Yao-Chia Shih, Kuan Jin Lee, Louis C. S. Tan, Eng King Tan, Ling Ling Chan

**Affiliations:** ^1^Department of Neurology, National Neuroscience Institute, Singapore, Singapore; ^2^Duke-NUS Graduate Medical School, Singapore, Singapore; ^3^Department of Diagnostic Radiology, Singapore General Hospital, Singapore, Singapore; ^4^Yuan Ze University, Taoyuan, Taiwan; ^5^Singapore BioImaging Consortium (SBIC), Agency for Science, Technology and Research (A*STAR), Singapore, Singapore

**Keywords:** Parkinson's disease, MRI, substantia nigra, classification, comparative study, correlation analysis, iron, neuromelanin

## Abstract

**Background:**

Neuromelanin- and iron-sensitive MRI studies in Parkinson's disease (PD) are limited by small sample sizes and lack detailed clinical correlation. In a large case–control PD cohort, we evaluated the diagnostic accuracy of quantitative iron–neuromelanin MRI parameters from the substantia nigra (SN), their radiological utility, and clinical association.

**Methods:**

PD patients and age-matched controls were prospectively recruited for motor assessment and midbrain neuromelanin- and iron-sensitive [quantitative susceptibility mapping (QSM) and susceptibility map-weighted imaging (SMWI)] MRI. Quantitative neuromelanin–iron parameters from the SN were assessed for their discriminatory performance in PD classification using ROC analysis compared to those of qualitative visual classification by radiological readers of differential experience and used to predict motor severity.

**Results:**

In total, 191 subjects (80 PD, mean age 65.0 years; 111 controls, 65.6) were included. SN masks showed (a) higher mean susceptibility (*p* < 0.0001) and smaller sizes after thresholding for low susceptibility (*p* < 0.0001) on QSM and (b) lower contrast range (*p* < 0.0001) and smaller sizes after thresholding for high-signal voxels (*p* < 0.0001) on neuromelanin-sensitive MRI in patients than in controls. Quantitative iron and neuromelanin parameters showed a moderate correlation with motor dysfunction (87.5%: 0.4< | r | <0.6, *p* < 0.0001), respectively. A composite quantitative neuromelanin–iron marker differentiated the groups with excellent performance (AUC 0.94), matching the diagnostic accuracy of the best-performing reader (accuracy 97%) using SMWI.

**Conclusion:**

Quantitative neuromelanin–iron MRI is associated with PD motor severity and matched best-performing radiological PD classification using SMWI, with the potential to improve diagnostic confidence in the clinics and track disease progression and response to neuroprotective therapies.

## Introduction

Parkinson's disease (PD) is a common neurodegenerative disease characterized clinically by rest tremor, bradykinesia, rigidity, and postural instability. Clinical features are only apparent after a significant (50–70%) loss of dopaminergic neurons in the substantia nigra (SN) (Sulzer et al., 2018). The clinical diagnosis of PD may be difficult (Rizzo et al., 2016; Beach and Adler, 2018), and a recent poll of PD patients reported misdiagnosis in 26%, and a further 21% saw a general practitioner three times before a specialist referral was made (Parkinson's UK, 2020). Non-invasive neuroimaging holds promise in improving confidence in the clinical diagnosis and management of PD. High-resolution neuromelanin- and iron-sensitive MRI of the midbrain are useful sequences in delineating radiological biomarkers in the SN in PD patients (Pavese and Tai, 2018; Pyatigorskaya et al., 2020; Cho et al., 2021a,b). However, the value of their quantitative markers is moot.

Human cadaveric studies have shown that neurons rich in the dark-brown cytoplasmic neuromelanin pigment in the midbrain are susceptible to degeneration in PD (Sasaki et al., 2006; Sulzer and Surmeier, 2013). The loss of dopaminergic neurons in these vulnerable neuromelanin-rich brain regions in the SN underlies the characteristic motor symptoms of the disease (Sasaki et al., 2006; Sulzer et al., 2018). *In vivo* neuromelanin-sensitive MRI reliably quantified nigral damage and distinguished PD from healthy subjects (Wang et al., 2019). Iron also plays an important role in the neurodegenerative process in PD; free iron promotes the production of toxic-free radicals leading to dopaminergic cell death (Dexter et al., 1989). Iron-sensitive MRI such as quantitative susceptibility mapping (QSM) has consistently found increased susceptibility in the SN of PD patients (Pyatigorskaya et al., 2020; Tan et al., 2021). Pathological correlates on ultrahigh field MRI support preferential dopaminergic cell loss in nigrosome-1, a main sub-component of the SN, as an accurate MRI biomarker in PD (Blazejewska et al., 2013; Sung et al., 2018; Bae et al., 2021a).

Although the literature suggests good results for differentiating PD patients from healthy controls using neuromelanin- and iron-sensitive MRI techniques, these studies are limited by small sample sizes, non-comparative single modal evaluations, inadequate discriminant reliability for clinical adoption, or lack of evaluation by readers of differential training and systematic or detailed clinical characteristics for robust correlation with imaging markers. To address these gaps, we conducted a large prospective case–control study to evaluate the diagnostic accuracy and clinical association of quantitative iron–neuromelanin parameters and compared these against a qualitative visual evaluation of nigrosome-1 and neuromelanin hyperintensity as proxies of nigral dopaminergic neurodegeneration across radiological readers of differential training. We hypothesized that quantitative iron–neuromelanin parameters can add value to the radiological workflow in the clinical evaluation and management of Parkinsonism, beyond a role in research settings.

## Materials and methods

### Subjects

This study was approved by the local ethics board, and informed consent was obtained from all participants. Patients were clinically diagnosed with PD by four movement disorders neurologists (two with 24, one with 15, and the last with 8 years of experience) using the Movement Disorder Society Clinical Diagnostic Criteria for Parkinson's disease (Postuma et al., 2015), and prospectively recruited from the clinics at our tertiary referral center. Only patients with clinically established PD were recruited. Age-matched healthy controls without neurological conditions were recruited from the community, health screening clinics and among the spouses of patients in the hospital clinics. Subjects with MRI contraindications, claustrophobia, known neurological/psychiatric diagnosis other than PD, chronic debilitating medical conditions, or poor cognitive function that would hinder patients' understanding of the study were excluded. All participants underwent clinical motor and non-motor assessments, including the Unified Parkinson's Disease Rating Scale motor sub-score (UPDRS-III), motor (UPDRS-II), and non-motor (UPDRS-I) aspects of experiences of daily living, and the Mini-Mental State Examination (MMSE). Demographic and clinical information were recorded in Table 1.

**Table 1:** Subject clinical demographics.

	**Healthy controls**	**PD patients**	***p*-value**
Number	111	80	
Age	65.6 ± 6.5	65.0 ± 9.3	0.66
Sex (M/F)	52/59	55/25	**0.003**
Motor			
Disease duration (years)	N.A.	6.7 ± 5.5	N.A.
Age of onset	N.A.	58.1 ± 11.4	N.A.
PD subtype	N.A.	44 Tremor 14 Bradykinesia 12 Rigidity 10 Mixed	N.A.
UPDRS-II	0.5 ± 1.7	7.8 ± 6.2	**< 0.0001**
UPDRS-III	3.4 ± 4.4	29.4 ± 13.3	**< 0.0001**
H&Y	0	2.0 ± 0.5	**< 0.0001**
LEDD	N.A.	452.9 ± 280.5	N.A.
Non-motor			
UPDRS-I	1.6 ± 2.9	5.6 ± 5.8	**< 0.0001**
MMSE	27.3 ± 2.1	25.9 ± 3.0	**0.0002**

Statistical significance is defined at *p* < 0.05 (bold). F, Female; H&Y, Hoehn and Yahr staging; LEDD, Levodopa equivalent daily dose; M, Male; MMSE, Mini-Mental State Examination; N.A., Not applicable; PD, Parkinson's Disease; UPDRS, Unified Parkinson's Disease Rating Scale.

### Image acquisition and processing

All subjects underwent brain MRI on a 3T scanner (Siemens Skyra, Erlangen, Germany). High-resolution midbrain sequences used the following MRI parameters: (1) 3D T2^*^ susceptibility weighted imaging (SWI) multi-echo gradient echo sequence (TR 48 ms, TE 13.77/26.39/39 ms, FA 20°, voxel size 0.5 × 0.5 × 1 mm^3^, slices 32, duration 4.15 min); (2) neuromelanin-sensitive T1 TSE sequence (TR/TE 938/15 ms, voxel size 0.5 × 0.5 × 3 mm^3^, slices 13, duration 10.42 min). The details of the standardized MRI scan planning protocol are found in Supplementary material and shown in Supplementary Figure S1. QSM and susceptibility map-weighted imaging (SMWI) images were reconstructed from the multi-echo GRE images using proprietary SMWI software (Seoul National University, Seoul, South Korea) (Nam et al., 2017). Quantitative susceptibility in parts per billion (ppb) was computed from QSM based on the STI Suite embedded in SMWI software (Li et al., 2014). Representative QSM, SMWI, and neuromelanin-sensitive MRI images in control and PD subjects illustrating the presence and absence of nigrosome-1 and substantia nigra hyperintensity, respectively, are shown in Supplementary Figure S1.

### Qualitative visual evaluation

**Figure 1:**
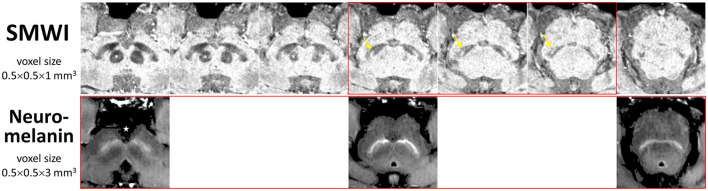
Anatomical landmarks for slice selection for qualitative visual evaluation and segmentation of the substantia nigra for quantitative image analysis. Corresponding consecutive (cranio-caudal—left-to-right) midbrain iron- (SMWI) and neuromelanin-sensitive MRI slices demonstrate the relations of the three slices (boxed in red) used between modalities. Loss of the normal hyperintense nigrosome-1 within the hypointense substantia nigra (yellow arrow) was assessed on SMWI slices just below the inferior pole of the red nucleus (asterisk). Loss of normal hyperintensity in the substantia nigra was assessed on neuromelanin-sensitive slices from the last slice through the interpeduncular fossa (star). Permission had been granted by the participating control subject for the publishing of these images.

SMWI and neuromelanin-sensitive anonymized MRI images of the first 80 study participants (47 PD patients and 33 healthy controls) were separately and independently reviewed by four radiologist readers of different training and experience (Table 2) using syngo.via (Siemens, Germany) on a clinical reporting workstation, blinded to the subject status. Readers 1, 2, and 3 had more than 2 decades, 1 decade, and only a year of neuroradiology practice, respectively, and Reader 4 had 6 years of non-neuroradiology practice. Reader 1 also completed a visual evaluation for the full cohort. Standardized slice selection (Figure 1) and the rating protocol were employed for PD classification, based on the visual assessment of a hyperintense nigrosome-1 substructure within the hypointense SN on SMWI images, and SN hyperintensity on neuromelanin-sensitive images, as detailed in Supplementary material.

**Table 2:** Classification performance of qualitative visual nigral evaluation by four radiologists using neuromelanin- and iron-sensitive (SMWI) MRI.

**MR**	**Reader**	**Reader 1–senior neuroradiologist (>20 years)**	**Reader 2–senior neuroradiologist (>10 years)**	**Average of Readers 1–2**	**Reader 3–junior neuroradiologist (1 year)**	**Reader 4–non-neuroradiologist (6 years)**	**Average of Readers 3-4**	***p*-value (Readers 1–2 vs. 3–4)**	**Average of all Readers**
Neuromelanin	Sensitivity	81%	68%	74.5%	100%	58%	79%	0.767	77%
	Specificity	93%	93%	93%	63%	59%	61%	**0.0002**	77%
	Accuracy	88%	83%	85.5%	78%	58%	68%	**0.008**	77%
SMWI (iron)	Sensitivity	100%	99%	99.5%	94%	74%	84%	0.086	92%
	Specificity	98%	96%	97%	89%	80%	84.5%	**0.026**	91%
	Accuracy	99%	97%	98%	91%	78%	84.5%	**0.005**	91%

Statistical significance is defined at a *p*-value of < 0.05 (bold).

### Quantitative analysis

SN masks (Supplementary Figure S2) and background regions of interest were manually drawn using MRIcroGL (University of South Carolina, USA) independently and separately by Readers 1 and 5 (postdoctoral researcher), blinded to subject status and using a standardized protocol (Figure 1), as detailed in Supplementary material. Quantitative MRI parameters from these, viz. susceptibility (QSM) or signal intensity (SMWI, neuromelanin-sensitive) values, and mask sizes, were extracted. The entire operational workflow from bilateral SN segmentation to quantitative parameter extraction was completed within 10 min on average per subject.

### Statistical analysis

Statistical analysis was performed using RStudio. Group comparisons were performed using chi-squared tests for categorical variables and two-tailed *t*-tests, or Kruskal–Wallis rank sum tests for continuous variables. Inter-rater agreement for qualitative visual evaluation was assessed using Fleiss' and Cohen's kappa. Inter-rater reliability of quantitative measures extracted from SN masks was assessed by intraclass correlation coefficient (ICC). Qualitative visual evaluation and quantitative SN parameters from Reader 1 were used for full cohort analysis. Receiver operating characteristic (ROC) analysis was performed to evaluate (a) the discriminative reliability of SMWI and neuromelanin-sensitive MRI in qualitative visual evaluation across readers and (b) the sensitivity and specificity of quantitative parameters as screening diagnostic discriminant tools between groups. Bivariate association between quantitative MRI parameters which were highly discriminant (AUC > 80%), and clinical parameters was performed using Spearman's correlation. A default statistical significance level was set at a *p*-value of < 0.05, and Bonferroni correction was performed for multiple comparisons.

## Results

### Clinical characteristics

Four subjects were excluded because of incomplete scans due to claustrophobia or poor image quality due to motion. A total of 191 subjects, comprising 80 PD patients and 111 age-matched healthy controls were finally included. Table 1 details the full clinico-demographics, and motor and non-motor characteristics of the study subjects. The patients had mild bilateral disease (mean UPDRS-III scores <33) without impaired balance, and the majority were of the tremor-dominant subtype (Table 1).

### Qualitative visual evaluation

Image quality was good or acceptable for all included subjects. Inter-rater agreement was substantial for SMWI (Fleiss' κ = 0.743) and fair for neuromelanin-sensitive MRI (κ = 0.366) across all Readers 1–4. For senior Readers 1–2, this was excellent for SMWI (Cohen's κ = 0.922) and substantial for neuromelanin-sensitive MRI (κ = 0.634).

PD classification performance was variable across Readers 1–4 for SMWI (sensitivity 74–100%, specificity 80–98%, and accuracy 78–99%) and neuromelanin-sensitive (58–100%, 59–93%, and 58–88%) MRI but superior among senior Readers 1-2 (Table 2).

### Quantitative analysis

The quantitative neuromelanin, QSM, and SMWI parameters extracted from SN masks segmented by Readers 1 and 5 showed excellent ICC (0.88, 0.95, and 0.97). ICC was also excellent (0.95) for area of SN masks segmented on SMWI and QSM. Group differences, effect sizes, and classification performance for quantitative parameters are detailed in Table 3.

**Table 3:** Group differences in quantitative neuromelanin- and iron-sensitive (QSM and SMWI) MRI from substantia nigra masks.

**MRI**	**Substantia nigra mask parameter**	**Healthy controls (mean ±sd)**	**PD Patients (mean ±sd)**	***p*-value**	**Hedges' g**	**AUC (95% CI)**
Neuromelanin	Signal intensity (a.u.)	226.8 ± 36.6	211.2 ± 54.2	0.091	0.348	0.58 (0.50–0.68)
	Contrast range (ratio of 90 to 10% signal intensity)	1.18 ± 0.12	1.10 ± 0.25	**< 0.0001**	0.431	0.86 (0.80–0.92)
	Size (mm^2^)	556.8 ± 108.1	580.34 ± 184.4	0.006	0.162	0.60 (0.52–0.68)
	Size after thresholding for high signal voxels (mm^2^)	92.3 ± 65.6	29.1 ± 30.6	**< 0.0001**	1.174	0.83 (0.77–0.89)
QSM (iron)	Mean susceptibility, χ (ppb)	84.5 ± 27.5	119.2 ± 33.5	**< 0.0001**	1.151	0.80 (0.74–0.86)
	Size (mm^2^)	277.7 ± 45.9	240.6 ± 56.7	**< 0.0001**	0.731	0.70 (0.63–0.78)
	Size after thresholding for voxels χ < 70 ppb (mm^2^)	102.3 ± 55.9	40.1 ± 42.1	**< 0.0001**	1.229	0.84 (0.78–0.90)
SMWI (iron)	Signal intensity (a.u.)	171.2 ± 49.7	145.9 ± 35.7	**< 0.0001**	0.570	0.67 (0.61–0.76)
	Size (mm^2^)	277.7 ± 45.9	240.6 ± 56.7	**< 0.0001**	0.731	0.70 (0.64–0.78)
	Size after thresholding for low signal voxels (mm^2^)	46.2 ± 70.4	83.2 ± 76.6	**0.0006**	0.506	0.79 (0.70–0.88)

Statistical significance is defined at a *p*-value of < 0.005 (bold) after Bonferroni correction. a.u., arbitrary units; AUC, area under the ROC curve; CI, confidence interval; ppb, parts per billion; sd, standard deviation.

SN masks on neuromelanin-sensitive MRI were more hyperintense (*p* = 0.091) and contained a higher (*p* < 0.0001) contrast range (ratio of 90th to 10th percentile signal intensity) in controls than in patients. These masks were three times smaller (*p* < 0.0001) in patients after optimized thresholding for high signal voxels (Supplementary Figure S2). Good PD classification performance was seen with both contrast range (sensitivity 80.2%, specificity 84.1%, and AUC 0.86) and smaller SN mask size after optimized thresholding for high signal voxels (79.3%, 74.4%, and 0.83).

QSM masks contained a higher (*p* < 0.0001) mean susceptibility (iron) in patients than in controls. These were 13% smaller (*p* < 0.0001) in patients, and the difference was magnified 4.6 times (*p* < 0.0001) after thresholding for voxels with low susceptibility (<70 ppb). The latter yielded good classification performance (sensitivity 76.6%, specificity 81.7%, and AUC 0.84). SMWI masks were darker and smaller (*p* < 0.0001) in patients than controls but were 1.8 times larger (*p* = 0.0006) in patients upon optimized thresholding for low-signal voxels. The latter showed decent PD classification performance (sensitivity 65.6%, specificity 78.7%, and AUC 0.79).

Based on the above findings, we derived a composite neuromelanin–iron marker using the product of the best-performing quantitative SN mask parameters (Supplementary Figure S3), viz. (i) neuromelanin contrast range, and mask sizes after optimized thresholding for voxels with (ii) high neuromelanin signal, and (iii) low susceptibility on QSM:


$$\begin{array}{l} \;Composite\;Marker\;=\\ \frac{Neuromelanin_{90th}}{Neuromelanin_{10th}}{\;}^{*}Size\;Neuromelanin_{high}{\;}^{*}Size\;QSM_{low} \end{array}$$

**Figure 2:**
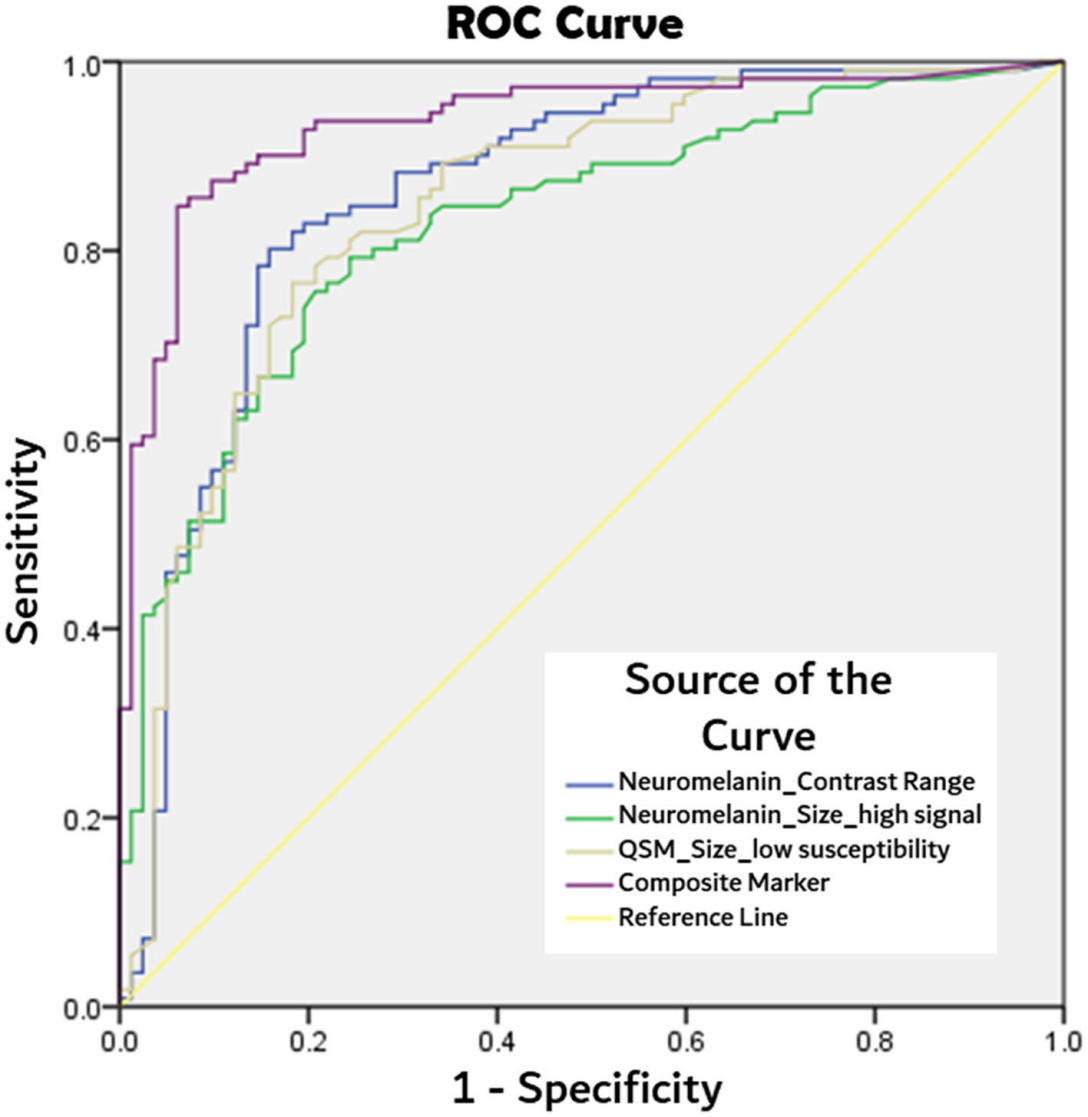
Comparison of receiver operating characteristics (ROC) curves from best performing quantitative MRI parameters of: neuromelanin signal contrast range within segmented substantia nigral mask, substantia nigra mask size after thresholding for high neuromelanin signal voxels, and low susceptibility (<70 ppb) voxels on QSM, and composite marker (product of three aforesaid parameters) in PD classification.

ROC analyses, as detailed in Table 4, showed the composite marker as the best-performing among quantitative parameters (Figure 2). Its AUC of 0.94 (at cut-offs: sensitivity 85%, specificity 94%, and accuracy 90%) compared favorably to qualitative PD classification (Reader 1) using SMWI (sensitivity 99%, specificity 95%, and accuracy 97%) and neuromelanin-sensitive MRI (79, 93, and 87%).

**Table 4:** Comparison of classification performance between qualitative visual and quantitative substantia nigra (SN) parameters using iron- and neuromelanin-sensitive MRI.

**MRI**	**MRI**	**SN feature**	**Accuracy**	**AUC (95% CI)**
Qualitative visual categoric	SMWI (iron)	Nigrosome-1	97%	N.A.
	Neuromelanin	Signal intensity and size	87%	N.A.
Quantitative from substantia nigral masks	Neuromelanin	Contrast range (ratio of 90 to 10% signal intensity)	82%	0.86 (0.80–0.91)
		SN mask size after thresholding for high signal voxels (mm^2^)	76%	0.83 (0.76–0.88)
	QSM (iron)	SN mask size after thresholding for voxels χ < 70 ppb (mm^2^)	80%	0.84 (0.79–0.90)
	Composite marker product of neuromelanin and iron parameters	$\frac{Neuromelanin_{90th}}{Neuromelanin_{10th}}$ *Size Neuromelanin_*high*_ *Size QSM_*low*_	90%	0.94 (0.90–0.97)

AUC, area under the ROC curve; CI, confidence interval; N.A., Not applicable.

### Association between clinical and quantitative MRI parameters from SN masks

On neuromelanin-sensitive MRI, segmented SN mask size negatively correlated with age (*r* = −0.22, *p* = 0.002). Multiple significant correlations were found between clinical motor and non-motor scores and quantitative neuromelanin and QSM parameters, which survived correction for multiple comparisons (Table 5). Specifically, there were moderate associations (87.5%: 0.4 < | r | < 0.6) between motor scores (UPDRS-II and UPDRS-III) and various neuromelanin and QSM parameters. There were also significant but weaker correlations between worse non-motor (higher UPDRS-I) scores and narrower neuromelanin contrast range and higher iron and smaller SN masks on QSM after thresholding. Additionally, cognitive impairment (low MMSE) was also associated with higher iron and smaller SN masks on QSM after thresholding. In the subset correlation analysis of PD patients alone, only QSM mask size after thresholding for low susceptibility correlated with MMSE (*r* = 0.306, *p* = 0.006) after Bonferroni correction.

**Table 5:** Association between clinical and quantitative MRI parameters from SN masks.

**MRI**	**Substantia Nigra Mask Parameter**	**Age**	**MMSE**	**UPDRS-I**	**UPDRS-II**	**UPDRS-III**
		**Coefficient r (** ***p*** **-value)**				
Neuromelanin	Contrast range (ratio of 90% to 10% signal intensity)	−0.051 (0.487)	0.196 (0.008)	**−0.274 (< 0.001)**	**−0.466 (< 0.001)**	**−0.532 (< 0.001)**
	Size after thresholding for high signal voxels (mm^2^)	–**0.220 (0.002)**	0.126 (0.087)	−0.173 (0.018)	**−0.372 (< 0.001)**	–**0.417 (< 0.001)**
QSM (iron)	Mean susceptibility, χ (ppb)	0.027 (0.713)	–**0.273 (< 0.001)**	**0.298 (< 0.001)**	**0.453 (< 0.001)**	**0.443 (< 0.001)**
	Size after thresholding for voxels χ < 70 ppb (mm^2^)	0.012 (0.865)	**0.272 (< 0.001)**	–**0.360 (< 0.001)**	–**0.479 (< 0.001)**	–**0.445 (< 0.001)**

Statistical significance is defined at a *p*-value of < 0.0025 (marked in bold) after Bonferroni correction (0.05/20). a.u., arbitrary units; MMSE, Mini-Mental State Examination; PD, Parkinson's Disease; UPDRS, Unified Parkinson's Disease Rating Scale.

## Discussion

This is a large case–control PD cohort study with complete clinico-imaging data, assessing the value add of quantitative multimodal neuromelanin–iron MRI parameters of the midbrain for screening and diagnosis, their clinical associations, and comparative performance against radiological qualitative evaluation across readers of varied experience and training. Our senior neuroradiology readers excelled in visual PD classification using SMWI, and this was superior to using unprocessed neuromelanin-sensitive MRI for both accuracy and inter-rater agreement. Reader experience affected visual classification performance whether SMWI or neuromelanin-sensitive MRI was used. Unique information from quantitative neuromelanin and iron (QSM) parameters in the segmented SN masks discriminated the groups with excellent performance (AUC 0.94) when harnessed in composite neuromelanin–iron marker and showed robust association with age, motor, and non-motor severity. Its disease classification performance also matched with that of senior neuroradiologists using SMWI in qualitative visual evaluation. Quantitative neuromelanin–iron MRIs are complementary techniques that provide high diagnostic accuracy and hold promise to more objectively aid PD diagnoses that are accurate, timely, and cost effective on a wide scale in the clinic.

Qualitative visual evaluation of nigrosome-1 on SMWI by senior neuroradiologists (Readers 1 and 2) showed excellent diagnostic accuracy (98%) for PD classification (Sung et al., 2019, 2022) and fared better than that in the literature using other iron-sensitive methodologies or quantitative analysis (Cho et al., 2021b). Diagnostic accuracy using unprocessed neuromelanin-sensitive MRI by our senior neuroradiology readers (85.5%) also compared favorably to that reported by small studies using qualitative visual evaluation, with or without thresholding for voxels with high signal (Pyatigorskaya et al., 2018; Cho et al., 2021a). Overall, our results and the literature suggest that visual evaluation of the midbrain for PD classification by radiologists using SMWI yielded the best performance. Between techniques, qualitative evaluation for loss of nigrosome-1 using iron-sensitive techniques provided better results than evaluation for loss of hyperintensity on neuromelanin-sensitive MRI as was also reported by the few small studies with cross-modal evaluation (Pyatigorskaya et al., 2018; He et al., 2021).

The superior sensitivity of SMWI is related to the ease of identification of the hyperintense nigrosome-1 within the iron-rich hypointense SN, making it an ideal visual tool for quick radiological reading. SMWI are multi-echo iron-sensitive post-processed images that combine local susceptibility from phase information and SWI magnitude images to improve the delineation of nigrosome-1 on clinical 3T scanners (Nam et al., 2017). SMWI heightens the contrast between voxels containing high and low susceptibility, sharply demarcating the SN margins and depicting the small nigrosome-1 substructure precisely within. The lower specificity of SMWI, and other iron-sensitive methodologies in the literature, compared to its supreme sensitivity in qualitative nigrosome-1 evaluation (Pyatigorskaya et al., 2018; Zorzenon et al., 2021; Sung et al., 2022), could be related to the occasional instance of an intact but tiny nigrosome-1 substructure, which was read as partially lost. In contrast, the posterolateral to the anteromedial gradient of depigmentation in the SN from neurodegeneration related to aging and PD (Supplementary Figure S2) is visually problematic on neuromelanin-sensitive MRI (Table 2) when incomplete. Image postprocessing through thresholding for high-signal voxels within the SN mask could assist in qualitative visual evaluation (Supplementary Figure S2).

Our quantitative analysis showed SN atrophy in PD patients compared to controls on iron-sensitive images (*p* < 0.0001, Table 3). On neuromelanin-sensitive MRI, the hyperintense SN margins were less distinct for patients (Supplementary Figure S2) and manifested as counter-intuitively larger SN masks (insignificant after Bonferroni correction, Table 3). Table 3 also shows how optimized thresholding for voxels with high-signal intensity (neuromelanin surrogate) or low susceptibility and SMWI signal (iron surrogate) helped accentuate volumetric differences in the dopaminergic midbrain neurodegeneration between groups (Kim et al., 2018; Cho et al., 2021a). Even though neuromelanin has a high binding affinity for iron, no significant correlation between NM and iron content in the SN has been reported (Reimão et al., 2016). This could account for the additive value of combined quantitative neuromelanin–iron MRI in a high-resolution protocol for midbrain evaluation in PD (He et al., 2021).

The composite quantitative neuromelanin–iron MRI marker achieved excellent classification performance (AUC 0.94) akin to the best qualitative visual evaluation using SMWI (97% accuracy, Table 4). This is important as SMWI is not widely available even though its diagnostic performance has been exceptional. Other image acquisition and post-processing techniques generating images with similar contrasts to SMWI (He et al., 2021; Liu et al., 2021) have reported lower classification performances (AUC 0.891–0.91) as spatial resolution (SMWI 0.5 × 0.5 × 1 mm^3^) affects the classification performance of the tiny nigrosome-1 as a radiological marker (Cho et al., 2021b; He et al., 2021; Liu et al., 2021). QSM images are readily reconstructed from multi-echo gradient echo sequences using open-source software (https://github.com/mathieuboudreau/qsm-tools). Adding a second neuromelanin-sensitive sequence to the MRI protocol prolongs total scan time and requires patient cooperation. Fortunately, recent multiband technology incorporated into neuromelanin-sensitive (standard T1 true spin echo) MRI acquisitions significantly reduces scan times (from 10 to 3 min).

The composite quantitative neuromelanin–iron marker has the potential for objective wide-scale diagnostic evaluation of Parkinson's disease through ease of accessibility and implementation in the clinical workflow. In our workflow, quantitative parameters from SN masks drawn by non-radiologist assistants were efficiently extracted in our image post-processing pipeline, with results made available in <10 min. While this could benefit non-expert readers the most, experienced readers could also use the additional data point to assist in the evaluation of borderline cases, with potential impact on clinical management (e.g., when to commence levodopa treatment; titrating levodopa dose). Clinical evaluation and MRI qualitative visual evaluation of the SN are not always straightforward, particularly in the presence of vascular co-morbidities or early PD, respectively (Reimão et al., 2016; Beach and Adler, 2018; He et al., 2021). More confident diagnoses could reduce costs by screening and triaging only clinico-radiologically borderline or atypical cases of parkinsonism for further radionuclide dopamine transporter imaging evaluation. The latter is more expensive, invasive, less available in some parts of the world, and incurs a radiation dose penalty. This is relevant to growing numbers of patients with parkinsonian syndrome in rapidly aging populations.

Our large case–control cohort demonstrated multiple moderate correlations between motor dysfunction (higher UPDRS-II and III scores) and SN iron (QSM mean susceptibility), narrower neuromelanin contrast range, and smaller SN masks after optimized thresholding on both QSM and neuromelanin-sensitive MRI (Table 5). Physiological neurodegeneration and nigral pathology increase with age, and mild parkinsonian signs are also common in elders without PD (Louis and Bennett, 2007; Buchman et al., 2012; Aye et al., 2020). The inclusion of a considerable number of control subjects who also underwent full clinical assessments in our cohort could explain our robust findings on correlation analyses. In contrast, individual small studies have reported limited, weak, or lateralized correlation of contralateral motor score in PD using either iron-sensitive or optimized neuromelanin-sensitive MRI methodologies (Schwarz et al., 2011, 2017; Miyoshi et al., 2013; Wang et al., 2018; He et al., 2021) or pooled meta-analysis for improved correlations (Pyatigorskaya et al., 2020). Older subjects in our cohort had smaller SN masks on neuromelanin-sensitive MRI (*r* = −0.22, *p* = 0.002), suggesting a trajectory of depigmentation in the SN with advancing age. This negative association of SN neuromelanin-related hyperintensity with age agrees with a lifespan study that included a small cohort (*n* = 30) of elderly aged >60 years, using a suprathreshold volume based on neuromelanin-sensitive MRI signal (Xing et al., 2018). Postmortem histological studies (Ma et al., 1999) also observed volume reductions of the pigmented SN from middle age. In addition to concordance with dopamine transporter imaging, quantitative neuromelanin-sensitive MRI has been reported to be more effective as a predictor of motor fluctuations in advanced PD (Okuzumi et al., 2019).

We found fewer and weaker associations between non-motor (MMSE and UPDRS-I) scores and midbrain MRI parameters. This is not surprising as non-motor features in PD are also known to be related to extranigral multisystem neuropathology involving neurotransmitter pathways beyond dopamine deficiency (Sulzer and Surmeier, 2013). Taken together, the observed quantitative MRI correlations with clinical scores, as measures of parkinsonian motor and non-motor dysfunction across a continuum of nigral and related extranigral neurodegeneration, from normal aging to PD, indicate the potential of quantitative iron–neuromelanin MRI as objective tools to track dopaminergic denervation, iron toxicity, and PD progression. Neuromelanin is thought to protect neurons against oxidative damage by inactivating free radicals or chelating transition metals (Pyatigorskaya et al., 2020). The degeneration of dopaminergic neurons in the SN (Cassidy et al., 2019) and breakdown of neuromelanin is thus accompanied by an increase in iron content, leading to further oxidative damage which manifests as the progression of the disease. Therefore, it is expected that excess iron deposition within the SN also serves as a specific imaging biomarker for PD progression.

Our study has its limitations. First, our manual SN segmentation is limited to three representative slices, while fully or semi-automated segmentation may enable interrogation of the entire SN volume and be less prone to variability, particularly for neuromelanin-sensitive MRI images (Cho et al., 2021a). Nonetheless, our inter-rater agreement for quantitative neuromelanin-sensitive MRI, QSM, and SMWI measures extracted from the manually segmented SN masks by radiology and non-radiology readers was excellent and yielded positive correlation results. These indicate that our technique can be practicably performed in the real-world clinical setting, with confidence that the manual segmentations are reliable. Second, we used SMWI for qualitative visual evaluation when it is not widely available as an open-source post-processing software. However, SMWI has produced superior results in visual analyses and has been extensively validated against dopamine transporter imaging evaluation for concordance (Sung et al., 2019, 2022; Bae et al., 2021b). Third, we did not perform deep learning analysis to further improve the diagnostic performance of our quantitative analysis (Jung et al., 2022). This could be the work of a future large cohort study. Fourth, our non-motor clinical assessments were limited and inadequately framed for the wide scoping non-motor symptomatology that exists in PD, including rapid eye movement sleep behavior disorder, which would be relevant for future study in prodromal PD in relation to composite midbrain quantitative neuromelanin–iron parameters.

## Conclusion

Our large, cross-modal, case–control study in PD showed (i) moderate associations between quantitative iron–neuromelanin MRI and motor and non-motor dysfunction, (ii) composite quantitative neuromelanin–iron marker matching best-performing qualitative radiological PD classification using SMWI, and (iii) potential for these techniques to be adopted to improve diagnostic confidence, tracking of disease progression and response to neuroprotective therapies in both clinical and research settings.

## Data availability statement

The original contributions presented in this study are included in the article/Supplementary material, further inquiries can be directed to the corresponding author.

## Ethics statement

The studies involving humans were approved by SingHealth Centralised Institutional Review Board (CIRB). The studies were conducted in accordance with the local legislation and institutional requirements. The participants provided their written informed consent to participate in this study.

## Author contributions

SH: Data curation, Formal analysis, Investigation, Software, Writing—original draft. RC: Investigation, Validation, Writing—review & editing. TW: Investigation, Writing—review & editing, Validation. AT: Investigation, Software, Writing—review & editing. WL: Investigation, Supervision, Visualization, Writing—review & editing. PT: Investigation, Software, Writing—review & editing. CC: Investigation, Writing—review & editing. WH: Investigation, Writing—review & editing. WT: Investigation, Writing—review & editing. EL: Investigation, Writing—review & editing. KP: Investigation, Writing—review & editing. Y-CS: Investigation, Supervision, Writing—review & editing. KL: Methodology, Software, Supervision, Writing—review & editing. LT: Investigation, Writing—review & editing. ET: Conceptualization, Investigation, Methodology, Resources, Supervision, Writing—review & editing. LC: Conceptualization, Data curation, Formal analysis, Funding acquisition, Investigation, Methodology, Project administration, Resources, Software, Supervision, Validation, Visualization, Writing—review & editing.
