# Supplementary material for: Quantitative iron–neuromelanin MRI associates with motor severity in Parkinson's disease and matches radiological disease classification
Source: Front Aging Neurosci. 2023 Nov 27;15:1287917. doi: 10.3389/fnagi.2023.1287917 (PMC10711072; doi:10.3389/fnagi.2023.1287917)
Supplement: Supplementary file 1 [file Data_Sheet_1.DOCX]

**Supplementary Materials**

*Image acquisition*

To enhance visualization of nigrosome-1 in the substantia nigra, the scan plane was aligned perpendicular to the midbrain in an oblique coronal plane.^1^ This same oblique-coronal scan plane was used for both the iron- and neuromelanin-sensitive MRI scan acquisitions. This was positioned perpendicular to the midbrain as symmetrically as possible using orthogonal scout images (Figure S1). Care was undertaken to ensure perpendicularity of the plane to the mid-sagittal line, and both hippocampi on the orthogonal coronal scout images through the pons. Each sequence was repeated with the patient on the table when significant motion was noted on visual inspection.

*Qualitative Visual* *Evaluation*

A standardized protocol, as previously described in the literature,^2,3,4^ was followed. Fixed window settings for image review were preset at 0-300 for SMWI and 800-1200 for neuromelanin-sensitive images. Three consecutive SMWI images from the slice just below the inferior pole of the red nucleus were evaluated for the presence of the hyperintense nigrosome-1 within the hypointense substantia nigra (Figures 1 and S1).^2^ Image reformatting on the fly (as would occur in clinical practice) was performed where needed for symmetry alignment to improve assessment (e.g., axial swallow-tail reformat parallel to the bicommisural line or orthogonal reformat to confirm inferior relations to the red nucleus. The SN on each side was rated as normal (clear visualization of a hyperintense nigrosome-1) or abnormal (complete or suspected loss of nigrosome-1). A subject was classified as normal only when both SN were rated normal.^2^ Neuromelanin-sensitive images (Figure S2) were classified as normal for a subject when high signal intensity was seen bilaterally in the SN without volume loss, and abnormal when reduced or loss of signal and/or size on one or both sides of the SN was seen.^3^ Readers 1-3 had experience with SMWI and neuromelanin-sensitive MRI visual evaluation and were comfortable with the neuroanatomical landmarks; Reader 4, a body radiologist, was only exposed to SMWI and neuromelanin-sensitive MRI and related neuroanatomical landmarks a week before the experiment.

*Quantitative Analysis*

The SN was manually segmented along its hypointense/hyperintense margins (depending on image contrast) on the same three slices selected for qualitative visual evaluation (Figure 1). The size of the SN masks and the following parameters were extracted using MATLAB (Mathworks, MA): mean signal intensity from SMWI and neuromelanin-sensitive masks, and mean susceptibility from QSM masks. A reference brainstem background circle region of interest (ROI) was also drawn to derive individual background mean values and standard deviation. Susceptibility and signal cutoffs to compute suprathreshold SN mask sizes optimized for PD classification were also determined, as previously described.^4,5^ SN mask sizes after optimized thresholding for voxels with high signal on neuromelanin-sensitive images (Figure S2),^5^ low susceptibility (<70 ppb) on QSM,^4^ and low signal on SMWI were tabulated in Table 3.

**References**

1. Nam Y, Gho SM, Kim DH, Kim EY, Lee J (2017) Imaging of nigrosome 1 in substantia nigra at 3T using multiecho susceptibility map-weighted imaging (SMWI). J Magn Reson Imaging 46:528-536
2. Sung YH, Kim JS, Yoo SW et al (2022) A prospective multi-centre study of susceptibility map-weighted MRI for the diagnosis of neurodegenerative parkinsonism. Eur Radiol. 10.1007/s00330-021-08454-z
3. Pyatigorskaya N, Magnin B, Mongin M et al (2018) Comparative Study of MRI Biomarkers in the Substantia Nigra to Discriminate Idiopathic Parkinson Disease. AJNR Am J Neuroradiol 39:1460-1467
4. Kim EY, Sung YH, Shin HG, Noh Y, Nam Y, Lee J (2018) Diagnosis of Early-Stage Idiopathic Parkinson's Disease Using High-Resolution Quantitative Susceptibility Mapping Combined with Histogram Analysis in the Substantia Nigra at 3 T. J Clin Neurol 14:90-97
5. Schwarz ST, Rittman T, Gontu V, Morgan PS, Bajaj N, Auer DP (2011) T1-weighted MRI shows stage-dependent substantia nigra signal loss in Parkinson's disease. Mov Disord 26:1633-1638

**Supplementary Figures**

**Figure S1.** Anatomical landmarks for planning high resolution 3D midbrain MRI and representative reconstructed iron- (QSM, SMWI) and neuromelanin-sensitive MRI images. (A) Using the mid-sagittal scout image, the oblique scan plane (yellow dotted line) is prescribed parallel to the line joining the red dots at the posterior commissure and superior border of pons (*left image*). The scan slab of the iron-sensitive (SWI multi-echo gradient echo) sequence is centered at the anterior pontomesencephalic junction (*center image*). The neuromelanin-sensitive scan follows, using the same scan angulation and first slice (red line) placement (*right image*). (B) Coned down QSM (l*eft*), SMWI (c*enter*) and neuromelanin (*right*) images illustrate the presence of nigrosome-1 (yellow arrows) and hyperintensity in the substantia nigra in a healthy elderly control subject (upper row) and loss of these imaging markers in an age-matched PD patient (lower row) respectively. Permission had been granted by the participating control subject for the publishing of these images.


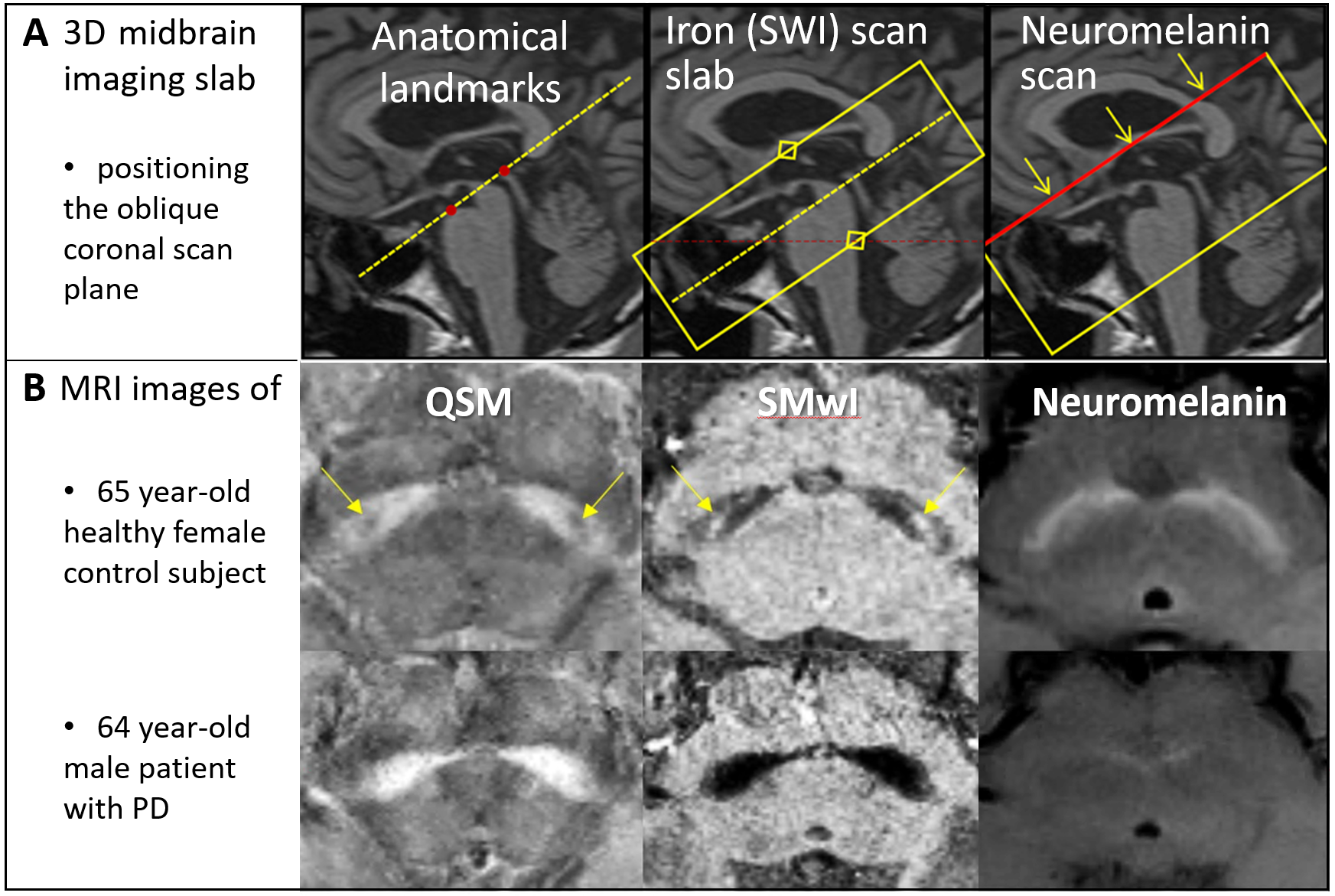


**Figure S2.** Optimizing diagnostic discrimination between groups by thresholding for high signal voxels within the substantia nigra using quantitative neuromelanin-sensitive MRI parameters. Compare the normal hyperintensity in the substantia nigra in an elderly control subject, resembling the front profile of outspread wings of a bird in flight (1^st^ row, left columns), to the gradient of signal loss in a posterolateral-anteromedial direction across the substantia nigra in a Parkinson’s disease patient (1^st^ image, right column). Systematic quantitative suprathreshold signal cutoffs (left rows) accentuate differences in the number of supra-threshold voxels remaining in the substantia nigra mask between the control (middle column) and Parkinson’s disease (right column) subjects. Abbreviations: b = signal intensity of the reference brainstem background region of interest, SI = signal intensity in each voxel, std = standard deviation in the background region of interest. Permission had been granted by the participating control subject for the publishing of these images.


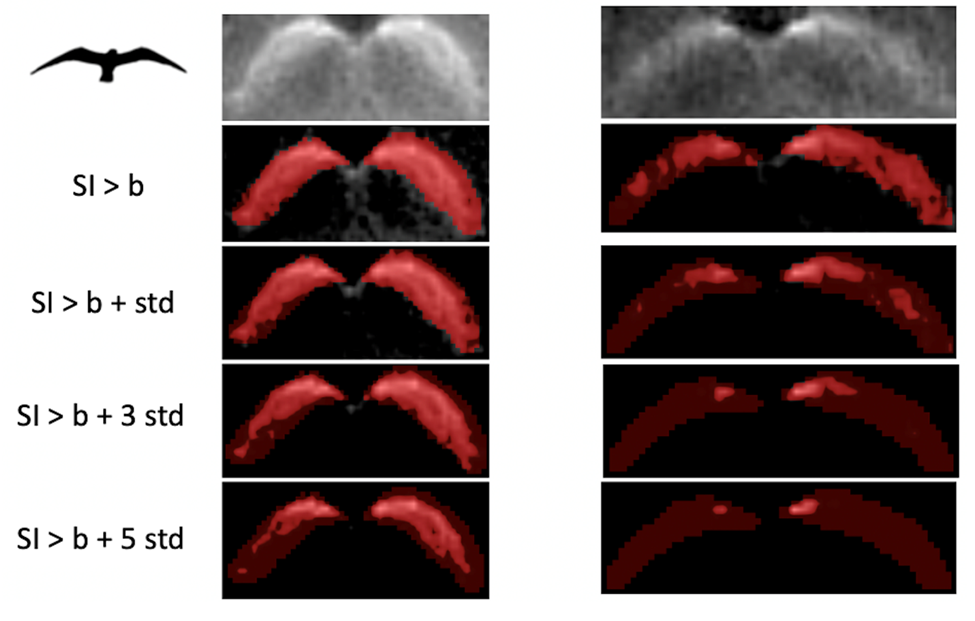


**Figure S3.** Group differences between Parkinson’s disease (PD) patients and healthy controls using quantitative MRI parameters from substantia nigra masks: (A) contrast range of neuromelanin signal (ratio of 90th to 10th percentile signal intensity), (B) size after thresholding for high neuromelanin signal voxels, (C) size after thresholding for low susceptibility (<70 ppb) voxels on QSM, and (D) composite marker - product of three aforesaid quantitative parameters.

**
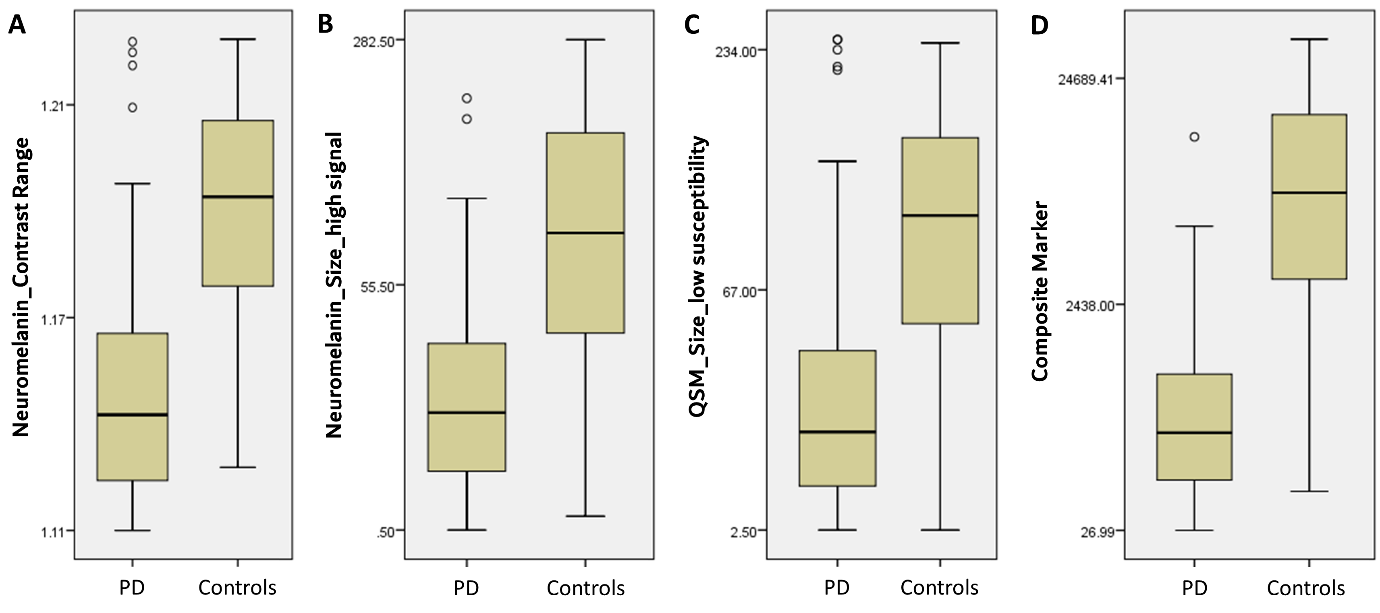
**
